# Trends in insecticide resistance in *Anopheles* mosquitoes (Diptera: Culicidae) in Ghana: a systematic review

**DOI:** 10.1093/jme/tjaf133

**Published:** 2025-10-09

**Authors:** Isaiah Debrah, Kassim A Rashid, Samuel K M Mensah, Evans K E Dormenyoh, Bismark Minnah, Fred Aboagye-Antwi, Yaw Aniweh, Gordon Awandare, Lucas N Amenga-Etego

**Affiliations:** West African Centre for Cell Biology of Infectious Pathogens (WACCBIP), College of Basic and Applied Sciences, University of Ghana, Accra, Ghana; West African Centre for Cell Biology of Infectious Pathogens (WACCBIP), College of Basic and Applied Sciences, University of Ghana, Accra, Ghana; West African Centre for Cell Biology of Infectious Pathogens (WACCBIP), College of Basic and Applied Sciences, University of Ghana, Accra, Ghana; West African Centre for Cell Biology of Infectious Pathogens (WACCBIP), College of Basic and Applied Sciences, University of Ghana, Accra, Ghana; West African Centre for Cell Biology of Infectious Pathogens (WACCBIP), College of Basic and Applied Sciences, University of Ghana, Accra, Ghana; Department of Animal Biology and Conservation Science, University of Ghana, Accra, Ghana; West African Centre for Cell Biology of Infectious Pathogens (WACCBIP), College of Basic and Applied Sciences, University of Ghana, Accra, Ghana; West African Centre for Cell Biology of Infectious Pathogens (WACCBIP), College of Basic and Applied Sciences, University of Ghana, Accra, Ghana; Department of Biochemistry, Cell and Molecular Biology, University of Ghana, Accra, Ghana; West African Centre for Cell Biology of Infectious Pathogens (WACCBIP), College of Basic and Applied Sciences, University of Ghana, Accra, Ghana

**Keywords:** insecticide resistance, mechanisms of resistance, Ghana, malaria zones

## Abstract

Malaria continues to be a major public health issue in Ghana, contributing significantly to hospital outpatient visits. Vector control remains central to malaria prevention; however, the growing resistance of malaria vectors to insecticides presents a major obstacle to control and elimination efforts. This review examined the evolution of insecticide resistance in Ghana from 2001 to 2024, summarising resistance mechanisms across the country’s bioclimatic zones to inform evidence-based vector control strategies aligned with Ghana’s malaria elimination goals. A systematic literature search was conducted using PubMed, Google Scholar, and Scopus databases to identify studies on insecticide resistance in major malaria vectors. A total of 41 articles were retrieved, and data were analysed using Microsoft Excel 365 and GraphPad Prism v.9.1.2. Pyrethroids were the most frequently studied insecticides, particularly in the Coastal (48%, *n* = 17), Forest (37.1%, *n* = 13), and Sahel (14.3%, *n* = 5) zones. An increasing trend of pyrethroid resistance in *Anopheles gambiae s.l*. was observed across all transmission zones, with the *vgsc*-L995F mutation being the most reported resistance mechanism. Temporal analysis revealed significant differences in resistance levels over time across all zones. Resistance to dual-active ingredients (piperonyl butoxide + pyrethroid) was also detected nationwide. Notably, there are limited studies on *An. funestus* susceptibility and metabolic resistance driven by copy number polymorphisms or *vgsc* variants. Given these gaps, the application of genomic surveillance and whole genome sequencing is essential for identifying locally relevant resistance mechanisms to guide future vector control interventions in support of Ghana’s malaria elimination efforts.

## Graphical abstract

**Graphical Abstract:**
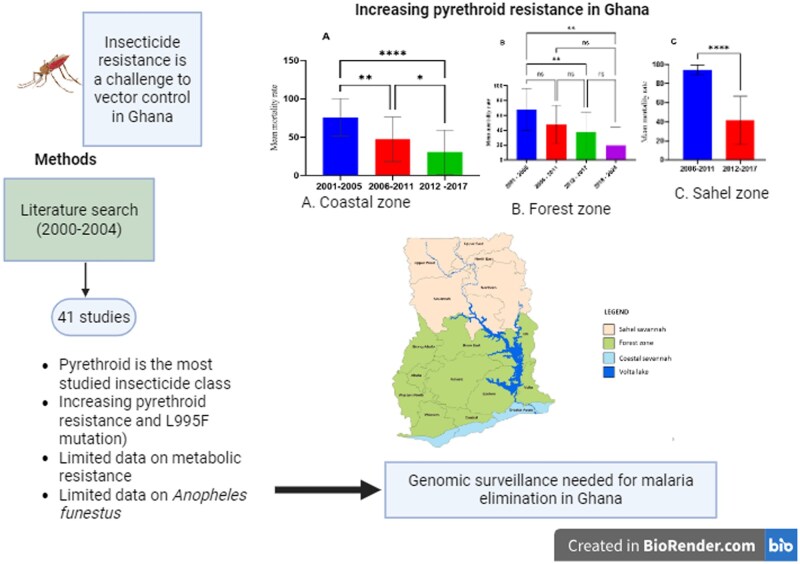

## Introduction

Malaria is a life-threatening disease caused by parasites of the genus *Plasmodium*, primarily transmitted to humans through the bites of infected female *Anopheles* mosquitoes, making vector control a cornerstone of global prevention strategies. The five species of *Plasmodium* that infect humans include *P. falciparum*, *P. vivax*, *P. malariae*, *P. ovale*, and *P. knowlesi*, with *P. falciparum* causing the most severe cases and fatalities, particularly in sub-Saharan Africa (Sinden and Gilles 2017). The World Health Organization (WHO) reported approximately 263 million malaria cases globally in 2023, with the African region accounting for about 94% of these cases and 95% of malaria-related deaths. Despite significant investments in malaria control over the past decade leading to the ramp-up of high-end vector control interventions such as indoor residual spraying (IRS) (Pluess et al. 2010), long-lasting insecticide-treated nets (LLINs) including dual active ingredient products (Dye et al. 2010, Accrombessi et al. 2021), coupled with rapid malaria diagnostic tests (RDTs) and artemisinin-based combination therapies (ACTs), malaria continues to be a leading cause of morbidity and mortality in endemic regions, owing to a dual genetic threat driven by the inherent ability of both malaria vectors and parasites to rapidly evolve resistance to front-line public health interventions (Ranson and Lissenden 2016, Shibeshi et al. 2020).

Insecticide resistance mechanisms underlying the phenotypic resistance have been classified into target site mechanism, metabolic mechanism, behavioural resistance and cuticle thickening to prevent or reduce the penetration of the insecticide (Hemingway 2000, WHO 2018). One of the most well-­characterized mechanisms of target-site resistance is the knockdown resistance (*kdr*) mutation, which affects the voltage-gated sodium channel (vgsc) gene (Martins et al. 2017). The vgsc is a key target for pyrethroids and DDT, which disrupt normal nerve function by keeping sodium channels open, leading to paralysis and death (Sharifnia 2024). Knockdown resistance is an example of the target site mechanism which occurs as a result of a non-synonymous amino acid substitution at position 995 of the vgsc protein leading to either the substitution of leucine for phenylalanine (Leu/Phe), also known as *kdr* west (L995F) (Martinez-Torres et al. 1998) or leucine for serine (Leu/Ser), known as *kdr east* (L995S) (Ranson et al. 2000). Both substitutions are characterized to confer cross-resistance to DDT and pyrethroids (Chandre et al. 1999).

Metabolic resistance is one of the most prevalent and significant mechanisms of insecticide resistance in mosquitoes. This type of resistance arises when mosquitoes over express or enhance the activity of specific detoxification enzymes that metabolize or sequester insecticides, reducing their effectiveness (Panini et al. 2016). These enzymes can prevent the toxicant from reaching its target site or neutralize it before it can exert its lethal effect. Key mechanisms of metabolic resistance include cytochrome P450 monooxygenases, glutathione s-transferases (GSTs), and esterases among others (Riveron et al. 2017).

**Fig. 1.**
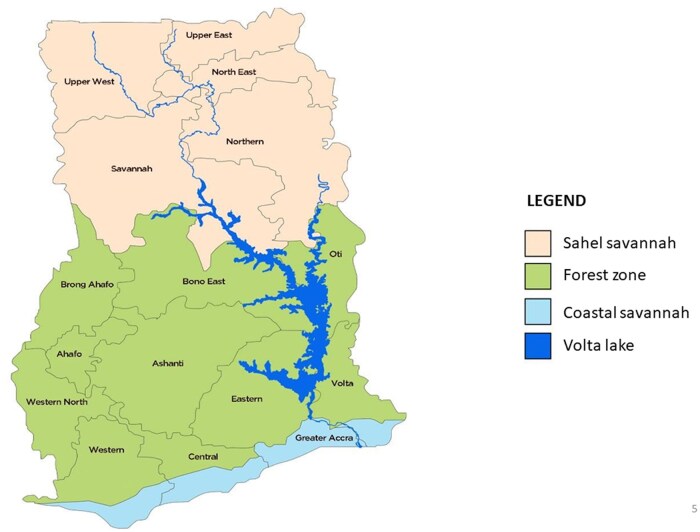
Map of Ghana showing the malaria epidemiological zones.

Ghana is among 11 nations that accounted for 70% of the global estimated malaria caseload and 71% mortality (WHO 2020). The whole Ghanaian population is at risk of malaria infection; transmission is heterogeneous and varies across natural epidemiological zones (Fenny and Akuffo-Henaku 2021). Malaria endemicity in Ghana is classified into three main zones namely; the coastal Savannah Zone, the forest transitional zone and the Sahel savannah zone (Dadzie et al. 2013, Amoah et al. 2021; Fig. 1). A secondary forest with grassland and small shrubs and high humidity characterizes the coastal zone. The coastal population is engaged in subsistence farming and fishing with the annual rainfall averages 350 to 1,200 mm (Dadzie et al. 2013). The forest transitional zone is predominantly deciduous, and the locals are mostly small to large-scale farmers and traders with significant mining activities. A thick network of rivers and streams characterizes the region, which receives roughly 2,000 mm of annual rainfall. The Sahel savannah zone receives an average of 850 mm of rainfall annually, most of which falls between May and October, with a long dry season (Koram et al. 2003, Nkrumah et al. 2014), large irrigation schemes underpinning vegetable and rice farming during the dry season. Across these epidemiological zones, malaria incidence is high but heterogeneous. The Ghana National Malaria Elimination Programme (NMEP) in partnership with key stakeholders has over the years implemented strategic interventions to drive down malaria parasite prevalence and disease incidence.

Key among these strategic measures is insecticide-based vector control interventions that for many decades were based on pyrethroids (Hemingway 2014). These include insecticide-treated nets (ITNs), primarily long-lasting insecticidal nets (LLINs) and indoor residual spraying (IRS) of insecticides. The gradual scale-up and increased coverage of LLINs has resulted in significant decreases in malaria morbidity and mortality in Ghana since 2000 (Afoakwah et al. 2018). However, the effectiveness of vector control measures is being threatened by rising insecticide resistance, particularly against pyrethroids (Pwalia et al. 2019, Akuoko et al. 2024). In response, the NMEP has gradually expanded the distribution of piperonyl butoxide (PBO)-pyrethroid dual nets and introduced new insecticides like pirimiphos-methyl (Acetalic) and Fludora fusion (deltamethrin/clothianidin) in response to growing reports of widespread pyrethroid-based insecticide resistance (Dadzie et al. 2017, Fongnikin et al. 2020).

In northern Ghana, the use of IRS for 7 years has contributed to a reduction in the malaria transmission intensity (Coleman et al. 2017). However, these gains are under threat due to malaria vectors across the world have developed resistance to at least one of the four classes of public health insecticides (organophosphates, carbamates, pyrethroids, and organochlorines) frequently utilized in vector control interventions (Ranson and Lissenden 2016, Hemingway 2018, WHO 2018). Pyrethroids and DDT target the voltage-sensitive sodium channel whereas organophosphate and carbamate inhibit the esterase resulting in the accumulation of acetylcholine in the synapses thereby impairing the nerve function and resulting in paralysis and death of the insect (Sparks and Nauen 2015).

Pyrethroid resistance is of particular concern, given this insecticide class is included in all World Health Organization (WHO) recommended LLINs and is also used for IRS in most countries. Resistance to pyrethroids has been considered a serious threat to malaria control programs (Hemingway 2018). According to the WHO's Global Technical Plan for Malaria 2016–2030 (WHO 2015), one of the primary biological obstacles to malaria control and elimination is malaria vector resistance to insecticides employed in core preventative strategies. It is against this background that the WHO launched the Global Plan for Insecticide Resistance Management (GPIRM) in 2012 (WHO 2012) for planning, executing and monitoring insecticide management at the national level, developing novel vector control tools, filling up the gaps in our understanding of resistance mechanisms and the impact of current resistance management strategies as well as enabling systems for better advocacy and the development of human and financial resources (WHO 2012, 2016).

Considering that the toolbox for vector control is limited, there is a need to assess the resistance status of the malaria vectors to current control tools, highlight the knowledge gaps to be addressed and propose how resistance can be managed in the malaria vector population. Understanding the evolution and the dynamics of insecticide resistance in Ghana could help in vector control interventions in the future. Using published research articles, we assessed the dynamics and trends of insecticide resistance of malaria vectors to public health insecticides and the mechanisms of resistance from the year 2000 to 2024 in the malaria ecological zones in Ghana.

## Materials and Methods

### Online Literature Search

An extensive search of published literature on insecticide resistance and mechanisms in malaria vectors using online bibliographic databases Google Scholar (http://scholar.google.com), PubMed (http://www.ncbi.nlm.nih.gov/pubmed), and Scopus (https://www.scopus.com/search/form.uri? display=advanced) was carried out. The key terms formulated and used to serve as a guide to search for the published literature in the databases included insecticide resistance, *Anopheles*, malaria vectors, susceptibility tests, insecticide resistance mechanisms and Ghana. The retrieved literature was examined, keeping all of the references that satisfied the following requirements for inclusion: (i) The article was published after December 2000 (from January 2001 to December 2024); (ii) the article reported primary data; (iii) the published article contained study site(s) in Ghana; (iv) insecticide resistance mechanisms and/or susceptibility testing were reported in the published article; (v) the published article reported the phenotypic resistance and mechanisms of resistance on *Anopheles* mosquitoes; (vi) the published articles followed the established WHO protocol for insecticide susceptibility testing (WHO 2016).

### Extraction and Curation of Data

The data were extracted into Microsoft Excel 365 data sheets and for each article, information extracted on phenotypic resistance and mechanisms included: Study sites (Ecological zone, village or town), authors(s) of the published article, year of publication, *Anopheles* species (*An. gambiae s.l.* and *An. funestus*), type of insecticide and insecticide class (pyrethroids, organophosphate, organochlorine, carbamate, and neonicotinoid), mortality rate of mosquitoes for each insecticide, and mechanism of resistance tested. The phenotypic resistance status in the vector population of the published articles was assessed based on the WHO threshold: susceptible, ≥98%; possible resistance, 90–97% and confirmed resistance, <90% (WHO 2016). The following was recorded for the mechanisms of resistance: type of knockdown resistance (*kdr*) mutations (vgsc-1014S, vgsc-1014F, vgsc-1575Y); allelic frequencies in percentage (%) and elevated activity of monooxygenases, glutathione S-transferases and carboxylesterase. The data obtained from the published articles were grouped under the malaria transmission zones (Coastal Savannah, Forest Zone, and Sahel Savannah) depending on reported study sites in the papers. We further grouped the research articles from each zone into year groups (2001–2005, 2006–2011, 2012–2017, and 2018–2024), the year in which the test was carried out as reported in the article. This was necessary to elucidate how insecticide resistance might have evolved in the zones. We observed that most published articles did not use molecular methods to distinguish sibling species of the *An. gambiae s.l.* and *An. funestus* groups, hence, we focused our analysis using the groups (*An. Gambiae s.l.* and *An. funestus*). We classified papers that reported on *Anopheles gambiae s.s.* and *An. coluzzii*, the main vectors that were reported in the articles as *An. gambiae s.l*. Conversely, *An. funestus* represents the *An. funestus* group.

### Data Analysis

The data analysis was performed using GraphPad Prism v. 9.1.2(226). One-way analysis of variance (ANOVA) was used to determine the mean differences in insecticide susceptibility of the various year group intervals (2001–2005, 2006–2011, 2012–2017, and 2018–2024) for each insecticide class in each epidemiological zone and Tukey multiple comparisons test was used to separate the means. Student *t*-test was used to compare the mean differences between two interval groups of the insecticide classes. Statistical significance was determined at *P* < 0.05. We could not find enough data points on *An. funestus* for comparison across the year groups in all the zones so we focused our analysis by comparing the susceptibility status between the forest zone and the Sahel savannah using a student *t*-test.

## Results

Our online searches yielded forty-one (41) articles published from 2001 to 2024 on insecticide resistance of malaria vectors in Ghana. Of these, 8 (19%) articles reported on the insecticide susceptibility tests only, 4 (10%) reported on resistance mechanisms only, and 29 (71%) reported on both insecticide susceptibility tests and mechanisms of resistance. Moreover, only one study reported on the intensity of insecticide resistance and five studies reported on the synergist-­insecticide resistance involving piperonyl butoxide (PBO) + insecticides, S, S, S,-tributyl-phosphorotrithioate (DEF) + insecticides and verapamil (VER) + insecticides in the vector population in Ghana. Our findings also revealed that most of the studies on susceptibility to insecticides and the mechanisms of resistance were conducted on *An. gambiae s.l.* (86%, *n* = 38) whereas *An. funestus* was the least studied species (14%, *n* = 6).

Most of the studies on the susceptibility status of *Anopheles* mosquitoes against public health insecticides were from the Coastal savannah zone (52.5%, *n* = 21). This was followed by the Forest zone (32.5%, *n* = 13) and the least being the Sahel Savannah (10%, *n* = 4) (Table 1). The same trend was observed in research articles that examined the resistance mechanisms in the vector populations. Seventeen (56.8%), 33.3%, *n* = 10 and 6.7%, *n* = 2 of papers reported mechanisms of resistance in the Coastal savannah, forest zone and Sahel savannah respectively (Table 1). Of all the research articles assessed, only one study examined the intensity of insecticide resistance, and six examined synergist + insecticide in the vector populations (Table 1).

**Table 1.** Reported insecticide susceptibility bioassays and resistance mechanisms of *Anopheles* mosquitoes in the ecological zones (2001–2024)

	Coastal savannah	Forest zone	Sahel savannah	All zones	Total (*N*)
	*n* (%)	*n* (%)	*n* (%)	*n* (%)	
**WHO insecticide resistance bioassay**	21 (52.5)	13 (32.5)	4 (10)	2 (5)	40
**Synergist-insecticide assays**	1 (17)	3 (60)	1(17)	1(17)	6
**Intensity bioassay**	1 (100)	0	0	0	1
**Resistance mechanism**	17 (56.7)	10 (33.3)	2 (6.7)	1 (3.3)	30

**Fig. 2.**
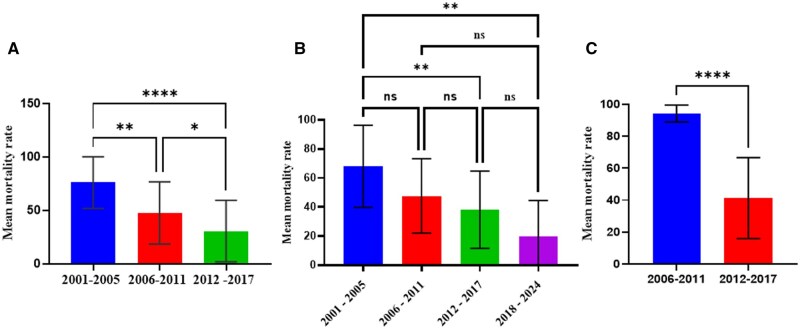
*An. gambiae s.l.* resistance to pyrethroids by epidemiological zone. Panel A Coastal zone, Panel B Forest zone, Panel C Sahel savannah. Ns: not significant, > 0.05 (nonsignificant), < 0.05 (* = significant), > 0.01 (** = significant), < 0.001 (*** = highly significant), < 0.0001 (**** = highly significant).

Pyrethroids were the most studied class of insecticide, as the majority of the research articles across the three zones reported on their efficacy against malaria vectors. Forty-eight per cent (*n* = 17), (37.1%, *n* = 13), and (14.3%, *n* = 5) of research articles reportedly assessed the susceptibility of *Anopheles* mosquitoes against pyrethroids in the Coastal, forest, and Sahel zones, respectively (Table 2). We observed a continuous increase in pyrethroid resistance in *An*. *gambiae s.l.* mosquitoes in all the malaria epidemiological zones in Ghana. One-way ANOVA showed significant differences among the year groups (*F* = 16.35; df = 2, 103; *P* < 0.0001) in the coastal zone, (Fig. 2A) and the forest zone (*F* = 5.55; df = 3, 79; *P* = 0.0017) (Fig. 2B). However, a significant difference in pyrethroid resistance was observed only between the 2006–2011 and 2012–2017-year groups in the Sahel zone (*t* = 4.150; df = 77; *P* < 0.0001) (Fig. 2C).

**Table 2.** Reported insecticide classes and resistance mechanisms of *Anopheles* mosquitoes by ecological zone

	Coastal savannah	Forest zone	Sahel savannah	Total (*N*)
	*n* (%)	*n* (%)	*n* (%)	
**Insecticide class**				
**Pyrethroids**	17 (48.6)	13 (37.1)	5 (14.3)	35
**Organochlorines**	9 (37.5)	12 (50)	3 (12.5)	24
**Organophosphates**	8 (38.1)	10 (47.6)	3 (14.3)	21
**Carbamates**	9 (42.9)	10 (47.6)	2 (9.5)	21
**Neonicotinoids**	0 (0)	0 (0)	1 (100)	1
**Insecticide resistance mechanisms**				
**Vgsc-L1014F (L995F)**	16 (55.2)	8 (27.6)	5 (17.2)	29
**Vgsc-1014S (L995S)**	2 (50)	1 (25)	1 (25)	4
**Vgsc-1575Y**	0 (0)	0 (0)	1 (100)	1
**Ace1-119S**	6 (75)	1 (12.5)	1 (12.5)	8
**Monooxygenases**	3 (37.5)	5 (62.5)	0 (0)	8
**Carboxylesterase**	1(25)	3 (75)	0 (0)	4
**Glutathione S-transferases**	1 (20)	3 (60)	1(20)	5

**Fig. 3.**
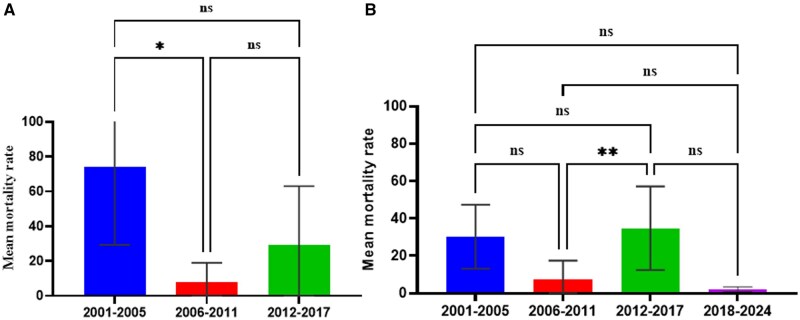
*An. gambiae s.l.* resistance to organochlorine in the malaria epidemiological zone in Ghana. A) Coastal Zone, B) Forest zone. No data for Sahel Savannah zone. Ns: not significant, > 0.05 (nonsignificant), < 0.05 (* = significant), > 0.01 (** = significant).

The forest zone had the highest number of published articles on organochlorine (50%, *n* = 12). This was followed by the coastal zone (37.5%, *n* = 9) and the Sahel savannah (12.5%, *n* = 3) (Table 2). *An. gambiae s.l.* resistance to organochlorine was observed in all the coastal and forest epidemiological zones. However, there were no significant differences in organochlorine resistance between year groups in the coastal (*F* = 5.213; df = 2, 20; *P* = 0.06) (Fig. 3A) and the forest zones (*F* = 5.360; df = 3, 25; *P* = 0.23) (Fig. 3B). There was insufficient data for organochlorine comparison between the year groups in the savannah zone.

**Fig. 4.**
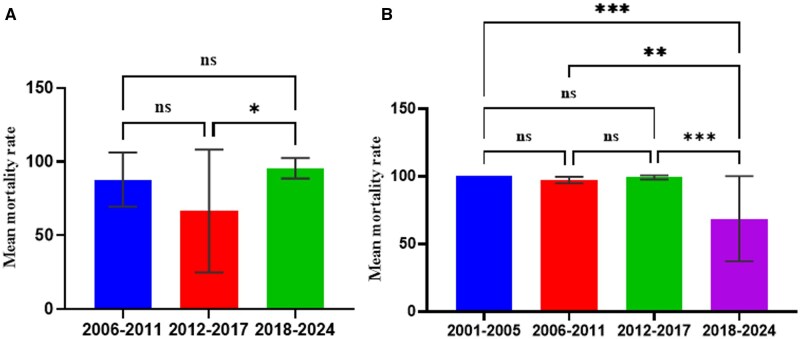
*An. gambiae s.l.* resistance to organophosphates in the malaria epidemiological zones in Ghana. A) Coastal Zone, B) Forest Zone Ns: not significant, > 0.05 (nonsignificant), < 0.05 (* = significant), > 0.01 (** = significant).

The forest zone had the highest number of research articles on *An. gambiae s.l.* susceptibility to organophosphate (47.6%, *n* = 10; Table 2). Moderate resistance to organophosphates was noticed in the coastal zone. However, the vectors were more susceptible to organophosphate in the forest zone for 2001–2005, 2006–2011, and 2012–2017 year intervals compared to 2018–2024 interval. (Fig. 4B). ANOVA analysis revealed no significant difference in organophosphate resistance in *An. gambiae s.l.* between the year groups in the coastal zone (*P* = 0.05) Fig. 4A. However, Tukey multiple comparison test showed a marginally significant difference in mean between the 2012–2017- and 2018–2024-year groups (*P* = 0.04) in the coastal zone (Fig. 4A). ANOVA analysis revealed a highly significant difference between the various year groups (*F* = 8.722; df = 3, 25; *P* = 0.0004) in the forest zone (Fig. 3B). Furthermore, Tukey multiple comparison showed a significant difference in organophosphate mortality rates between 2012–2017 and 2018–2024 (*P* = 0.0004) and between 2006–2011 and 2018–2024 (*P* = 0.0014) year groups in the forest zone (Fig. 4B).

**Fig. 5.**
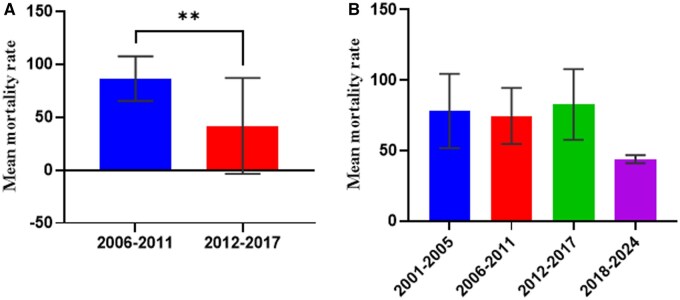
*An. gambiae s.l.* resistance to carbamate in malaria epidemiological zones in Ghana. A) coastal zone, B) Forest Zone, no data was found for Sahel Savannah zone.

Most studies on the *An. gambiae s.l.* susceptibility to carbamates was from the forest zone (47.6%, *n* = 10) (Table 2). *An. gambiae s.l.* has developed resistance to carbamate in coastal and forest zones in Ghana. There was a significant difference in the mean mortality rates of carbamate in *An. gambiae s.l.* between 2006–2011 and 2012–2017 year groups (*t* = 4.440; df = 41; *P* = 0.008) in the coastal zone (Fig. 5A). In contrast, there was no significant difference in *An. gambiae s.l.* resistance to carbamate between the year groups in the forest zone (*F* = 2.21; df = 3, 20; *P* = 0.118) (Fig. 5B). Studies on the *An. gambiae s.l.* susceptibility to carbamate in the Sahel zone are limited, hence, we could not find enough data points for the year-group comparisons.

Only one study assessed the efficacy of clothianidin (neonicotinoids) against *An. gambiae sl* (Table 2).

Vgsc-L1014F (L995F) target site mutation was the most studied mechanism of resistance in *An. gambiae s.l.* mosquitoes across all the zones (Table 2). Monooxygenases, carboxylesterase and glutathione S-transferases were the main gene families conferring metabolic resistance to insecticides in the vector populations in all the zones (Table 2).

**Fig. 6.**
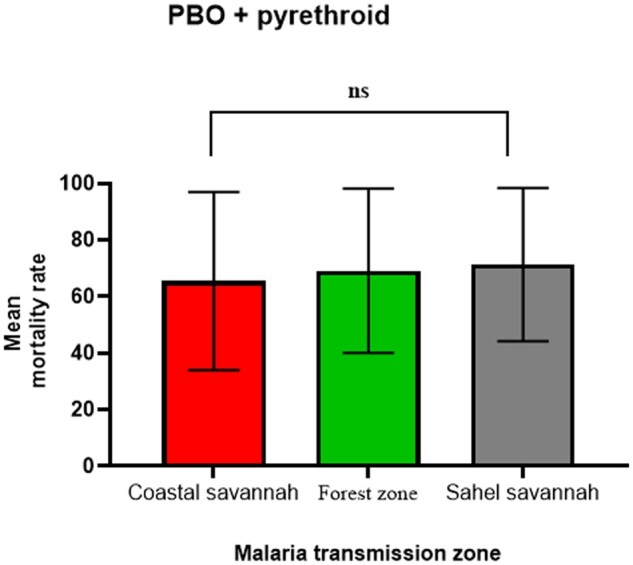
*An. gambiae s.l.* resistance to PBO + pyrethroids by epidemiological zones. Ns: not significant.

Studies involving susceptibility to the synergist (PBO) and pyrethroids were conducted from 2016 to 2022. These studies were conducted mainly on *An. Gambiae s.l.* mosquitoes. We could not find enough data points for the year groups to elucidate how the susceptibility status might have changed over time. We, therefore, compared the susceptibility status of the *An. gambiae s.l.* to the PBO+ pyrethroids between the zones. Our findings show that *An*. *gambiae s.l.* mosquitoes have developed resistance to the PBO+ pyrethroids in all the zones. ANOVA analysis revealed no significant difference between the three zones (*F* = 0.247; df = 2, 82; *P* = 0.78; Fig. 6).

**Fig. 7.**
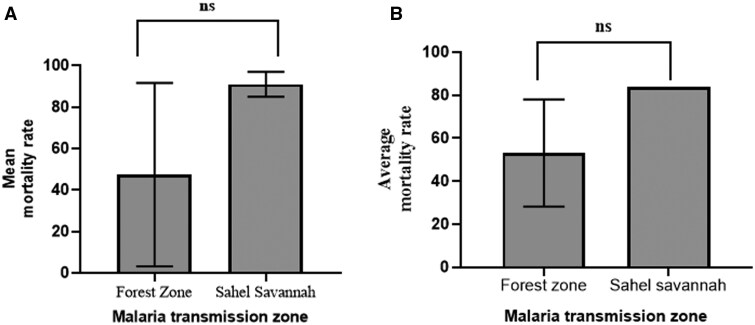
*An. funestus* resistance to insecticides in the forest and Sahel zones in Ghana. A) Pyrethroids, B) organochlorine, Ns: not significant.

Limited studies exist on the susceptibility status of *An. funestus* to insecticides in the malaria zones in Ghana. We obtained data on resistance in the *An. funestus* vector population in the forest and Sahel savannah zones, but one study assessed the susceptibility of *An. funestus* to PBO + pyrethroid in the forest zone. No further assessment of the intensity of insecticide resistance was reported. There was no significant difference between pyrethroid (*t* = 1.919; df = 12; *P* = 0.079) and organochlorine (*t* = 1.172; df = 7; *P* = 0.279) resistance in the forest and the Sahel savannah zones (Fig. 7).

## Discussion

Our findings have shown that from 2000 to 2024, insecticide resistance in malaria vectors has increased dramatically and rapidly spread across all the epidemiological zones in Ghana. This continuous decline in the efficacy of public health insecticides, particularly pyrethroids, has been of great concern in all malaria-endemic areas across sub-Saharan Africa (Mzilahowa et al. 2016, Ranson and Lissenden 2016, Hancock et al. 2018, Tungu et al. 2023). Pyrethroids are the main class of insecticides recommended for bed net impregnation due to their low mammalian toxicity, excito-repellency, and rapid knockdown effect, making them particularly effective for malaria vector control (Zaim et al. 2000).

Per our findings, *An*. *gambiae s.l.* susceptibility to pyrethroids has decreased and followed the same pattern in all the zones. The pattern is in order of 2001–2005 > 2006–2011 > 2012–2017 > 2017, 2001–2005 > 2006–2011 > 2012–2017 > 2018–2024, and 2006–2011 > 2012–2017 for the coastal, forest and Sahel zones respectively. This worrying trend of pyrethroid resistance in major vectors necessitated the incorporation of the synergist (PBO) in LLINs impregnation as dual active ingredient (PBO + pyrethroids) capable of inhibiting the activity of cytochrome P450 monooxygenases metabolic activity to enhance the efficacy of the pyrethroids (WHO 2015). Our findings have also shown, however, that *An. gambiae s.l.* has rapidly developed resistance to PBO + pyrethroids in all the zones of Ghana. This confirmed a recent study in the coastal zone in Ghana, where high resistance to PBO + pyrethroids was observed (unpublished data). A previous study has also noted that PBO-based bed nets could not solve the prevailing challenge of insecticide resistance in Southern Benin in *An. gambiae* and *C. quinquefasciatus* populations (N’Guessan et al. 2010). Moreover, a study reported an increasing pyrethroid resistance in *An. funestus* against PBO-based bed net (Riveron et al. 2019). This suggests that PBO-based bed nets may not effectively manage the prevailing insecticide resistance in the vector populations.

The use of new generation of insecticides including clothianidin, pyriproxyfen and chlorfenapyr, having new modes of action could be of great help in controlling notorious PBO + pyrethroid-resistant mosquitoes (Raghavendra et al. 2011, Zoungbédji et al. 2023). Moreover, the effectiveness of spatial repellents in controlling resistant malaria vectors has been reported (Ochomo et al. 2025). Incorporating these emerging tools into malaria control programs in Ghana could complement the existing tools and help reduce the risk of transmission.

*An. gambiae s.l.* has developed multiple resistance to organochlorine, organophosphate and carbamate insecticides across the malaria transmission landscape in Ghana. The rise in resistance to multiple insecticides in the vector population has been reported in other malaria-endemic areas (Yewhalaw et al. 2011, Nwane et al. 2013). The remarkable increase in insecticide resistance was attributed to the widespread use of pyrethroid-based aerosol sprays, mosquito coils and similar agricultural insecticides, which impose selection pressure on the vector population (Reid and McKenzie 2016, Chabi et al. 2018, Tungu et al. 2023).

The L995F mutation in the *vgsc* gene is the most characterized in the *An. gambiae s.l.* population across Ghana. Other studies have shown that this mutation has reached fixation in *An. gambiae s.l.* in West Africa (Kpanou et al. 2022, Ekedo et al. 2023, Namountougou et al. 2024). Our findings also revealed that few studies reported the presence of L995S and 1575Y mutations in Ghana. The main gene families responsible for resistance, as reported in the published articles, included monooxygenases, carboxylesterase and glutathione s-transferases. These studies used the microplate assay to analyse the nonspecific activity of the enzymes (Brogdon et al. 1988). The use of advanced technologies such as whole genome sequencing techniques could help to elucidate specific genes within the gene families and novel mutations conferring resistance in the vector populations. In general, the genomic architecture underlying the increase in phenotypic resistance in the *Anopheles* mosquito population in Ghana is poorly understood. Employing genomic tools to unravel the mechanisms of insecticide resistance could enrich our understanding and help to design effective control strategies.

*An. funestus* is known to be one of the major vectors of human malaria in Africa and contributes to over 80% of malaria transmission in areas where it dominates (Kaindoa et al. 2017, Mapua et al. 2022). However, there are limited studies on insecticide resistance and its contribution to malaria transmission in Ghana. It is commonly referred to as a minor vector, but our recent field surveys have shown that *An. funestu*s is more abundant than *An. gambiae s.l.* in certain areas in Ghana, including but not limited to the Eastern and Bono Regions of the forest zone, as well as Sissala East and Central Gonja Districts of the Savannah Zone, (unpublished data). Although in general, most of the published articles retrieved in this study on insecticide resistance were from the coastal zone, none reported resistance in *An. funestus* in the coastal zone of Ghana. The focal presence of *An. funestus* may be underpinned by micro-ecological variation leading to adaptation to specific environments that promote its survivability and efficiency as a malaria vector.

In general, this review reveals that limited past studies addressed insecticide resistance mechanisms and the intensity of resistance in vector populations in Ghana. Knowledge gaps exist in the susceptibility status of malaria vectors to various public health insecticides, including the new-generation insecticides.

## Conclusions

A continuous decline in the efficacy of public health insecticides, particularly pyrethroids, has been reported since 2001, when insecticide-based vector control was ramped up across Ghana. We have also noticed resistance to PBO + pyrethroids combinations against resistant malaria vector populations with limited information on the resistance mechanism in the vector populations. Incorporating new-generation insecticides with different modes of action and spatial repellents into the vector control toolbox could be the magic bullet for future malaria elimination in Ghana.
